# Biological Impact of Organic Extracts from Urban-Air Particulate Matter: An In Vitro Study of Cytotoxic and Metabolic Effects in Lung Cells

**DOI:** 10.3390/ijms242316896

**Published:** 2023-11-29

**Authors:** Tatiana D. Silva, Célia Alves, Helena Oliveira, Iola F. Duarte

**Affiliations:** 1 Department of Chemistry, CICECO—Aveiro Institute of Materials, University of Aveiro, 3810-193 Aveiro, Portugal; tatiana.silva@ua.pt; 2 Department of Biology, CESAM—Centre for Environmental and Marine Studies, University of Aveiro, 3810-193 Aveiro, Portugal; holiveira@ua.pt; 3 Department of Environment and Planning, CESAM—Centre for Environmental and Marine Studies, University of Aveiro, 3810-193 Aveiro, Portugal; celia.alves@ua.pt

**Keywords:** air pollution, PM_10_, polycyclic aromatic hydrocarbons, plasticizers, toxicometabolomics

## Abstract

Atmospheric particulate matter (PM) with diameters below 10 µm (PM_10_) may enter the lungs through inhalation and are linked to various negative health consequences. Emergent evidence emphasizes the significance of cell metabolism as a sensitive target of PM exposure. However, the current understanding of the relationship between PM composition, conventional toxicity measures, and the rewiring of intracellular metabolic processes remains limited. In this work, PM_10_ sampled at a residential area (urban background, UB) and a traffic-impacted location (roadside, RS) of a Portuguese city was comprehensively characterized in terms of polycyclic aromatic hydrocarbons and plasticizers. Epithelial lung cells (A549) were then exposed for 72 h to PM_10_ organic extracts and different biological outcomes were assessed. UB and RS PM_10_ extracts dose-dependently decreased cell viability, induced reactive oxygen species (ROS), decreased mitochondrial membrane potential, caused cell cycle arrest at the G0/G1 phase, and modulated the intracellular metabolic profile. Interestingly, the RS sample, richer in particularly toxic PAHs and plasticizers, had a greater metabolic impact than the UB extract. Changes comprised significant increases in glutathione, reflecting activation of antioxidant defences to counterbalance ROS production, together with increases in lactate, NAD^+^, and ATP, which suggest stimulation of glycolytic energy production, possibly to compensate for reduced mitochondrial activity. Furthermore, a number of other metabolic variations hinted at changes in membrane turnover and TCA cycle dynamics, which represent novel clues on potential PM_10_ biological effects.

## 1. Introduction

Particulate matter (PM), a major component of air pollution, encompasses a blend of solid particles and liquid droplets that exhibit diverse compositions and sizes. PM with diameters ≤10 μm (PM_10_) or ≤2.5 μm (PM_2.5_) are currently subject to regulation through ambient air quality standards, due to their well-established adverse health impacts [1]. The respiratory system is a major pathophysiological target of PM, with PM_10_ being inhalable into the lungs, where it can trigger inflammatory responses and tissue damage [2]. Moreover, the fraction of PM_10_ with smaller sizes, namely PM_2.5_, can penetrate deep into the airways, ultimately reaching the alveoli and infiltrating into the bloodstream [3]. Consequently, smaller particles have greater potential for harmful effects beyond the respiratory system, including renal, neurological, gastrointestinal, and reproductive systems [4]. Nevertheless, it is crucial not to underestimate the health impacts of the PM_10-2.5_ fraction (sizes between 2.5 and 10 µm), particularly concerning respiratory health [5]. Indeed, different studies have shown more pronounced pulmonary inflammatory effects for PM_10-2.5_ than for PM_2.5_, likely due to the preferential deposition of microbial products on coarse particles [6,7]. Hence, analysing the effects of PM_10_, which comprises smaller size ranges, represents a good proxy of the overall toxicity. Focusing on respiratory health problems, exposure to atmospheric PM has been linked to aggravation of respiratory conditions (e.g., asthma and chronic obstructive pulmonary disease) [8], lung cancer [9], and higher susceptibility to respiratory infections [10,11]. Moreover, it is becoming increasingly apparent that the interaction of PM with other major air pollutants—such as nitrogen dioxide and ozone—may influence health outcomes, particularly regarding cardiovascular and respiratory mortality [12,13].

PM-bound polycyclic aromatic hydrocarbons (PAHs) and plasticizers represent some of the most toxic organic components within PM [14,15,16]. Comprising multiple fused aromatic rings, PAHs predominantly originate from the incomplete combustion or pyrolysis of organic substances, including fossil fuels and biomass [17]. Once emitted into the atmosphere, PAHs undergo physical and chemical modifications, ultimately binding to airborne PM, an association that facilitates their transport and dispersion in the environment. Several PAHs, especially those with high molecular weights and a greater number of aromatic rings, have been classified as Group 1 human carcinogens by the International Agency for Research on Cancer (IARC) [18]. In addition to their size, the release rate of PAHs from PM and their uptake by cells, as influenced by factors like the microenvironmental pH [19], may also modulate their toxicity. Plasticizers, such as phthalates and adipates, are commonly used in the production of plastics and can be found in PM from both indoor and outdoor environments [20,21,22]. While representing a minor fraction of PM, some plasticizers are considered endocrine-disrupting chemicals [23] and/or have been associated with adverse effects on reproductive health and fetal development [24]. Moreover, some studies have suggested a potential link between phthalate exposure and respiratory problems, particularly in children [25]. 

The toxicity of airborne PM towards lung cells has been extensively investigated through in vitro tests. Commonly reported effects include diminished cell viability, induction of oxidative stress, inflammatory responses, and DNA damage [26,27,28,29,30]. Moreover, changes in cell metabolism have started to emerge as an important biological endpoint of PM exposure, mainly because (i) metabolic alterations tend to manifest at relatively low PM exposure concentrations, often preceding the detection of other traditional toxicity indicators; (ii) the dysregulation of metabolic processes induced by PM exposure may have significant implications for the pathobiology of various lung diseases [31]. However, our comprehension of how PM precisely modulates distinct metabolic pathways in diverse cell types remains limited. Furthermore, the relationship between these metabolic shifts and conventional toxicity markers remains inadequately elucidated. Further research is essential to bridge these knowledge gaps and deepen our understanding of the intricate interplay between PM exposure, cellular metabolism, and health outcomes.

By enabling a comprehensive and multiparametric assessment of variations in tens of metabolites simultaneously and with high reproducibility, untargeted metabolomics represents an exceptionally powerful tool for evaluating metabolic changes within cultured cells [32,33]. In recent years, metabolomics has been increasingly applied to investigate how lung cells respond to various PM samples, including certified reference materials [34,35] and environmental samples sourced from different locations, namely in China [36,37,38,39,40] and Spain [41,42]. The diverse array of findings arising from these studies underscores the dependence of metabolism modulation on PM features and exposure conditions, thus emphasizing the critical significance of incorporating metabolomic techniques into the biological evaluation of the effects exerted by PM.

The present study aims to characterize the biological effects of PM_10_ sampled at two different sites (urban residential background and roadside) of a mid-size Portuguese city by integrating untargeted metabolomics with conventional toxicity endpoints. The PM_10_ organic extracts were comprehensively characterized in terms of polycyclic aromatic hydrocarbons (PAHs) and plasticizers, given the well-documented high toxicity potential of these compounds. Human alveolar basal epithelial cells (A549 cell line) were used as an in vitro model to examine the effects on cell viability, generation of reactive oxygen species, mitochondrial membrane potential, cell cycle dynamics, and intracellular metabolic profile. Possible correlations between the different outcomes and PM_10_ extracts’ composition are discussed.

## 2. Results and Discussion

### 2.1. Composition of PM_10_ in PAHs and Plasticizers

**Figure 1:**
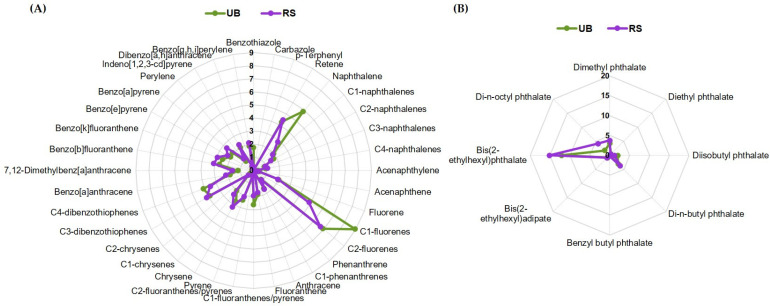
Relative amounts of individual (**A**) PAHs and (**B**) plasticizers in the UB and RS PM_10_ extracts, expressed as percentage of the total content of these compounds. For each compound, the masses present in the filter sections extracted and pooled together (for biological assays) were added up and divided by the total PAHs and plasticizers mass.

The total concentrations of PAHs and plasticizers in the PM_10_-loaded filters used in this work varied in the range of 133.9–483.9 ng/cm^2^. The detailed composition is provided in Supplementary Table S1. Based on these data, the concentrations of PAHs and plasticizers present in the pooled UB and RS samples prepared at an OC concentration of 25 µg/mL for biological assays were estimated to be 122.1 and 124.3 ng/mL, respectively. The UB sample contained a slightly higher concentration of PAHs (93.9 vs. 87.6 ng/mL), while plasticizers were higher in the RS sample (36.7 vs. 28.2 ng/mL). The relative contribution of each quantified compound to the total composition is represented in Figure 1. 

C1- and C2-fluorenes were the most abundant PAHs in both extracts (Figure 1A). Notably, these alkylated PAHs have been described as more toxic and persistent than their parent compounds [43]. Moreover, high-molecular-weight (HMW) 4- to 6-ring PAHs dominated over low-molecular-weight (LMW) 2- and 3-ring hydrocarbons, with HMW/LMW ratios of 2.3 and 3.8 for UB and RS, respectively. In particular, the RS sample presented higher concentrations of C2-chrysenes and 7,12-dimethylbenz[a]anthracene (1.4-fold difference), as well as of benzo[a]pyrene and perylene (1.3-fold difference). HMW PAHs are generally produced by the combustion of fossil fuels, biomass, and other organic materials, while LMW PAHs arise primarily from non-combusted petroleum products [44]. Hence, the abundance of HMW PAHs in our samples reflects the impact of pyrogenic sources, namely traffic emissions and residential wood burning. The higher HMW/LMW ratio for PAHs in the RS location is likely due to more intense traffic emissions with frequent braking. This difference may potentially have implications for toxicity, as, among other factors, the size of PAHs influences their carcinogenic and pro-oxidant potential [45]. Regarding plasticizers (Figure 1B), most compounds were more concentrated in the RS sample. The higher concentrations in this location may be associated with frequent braking at traffic lights and at the entrance to the nearby roundabout and the consequent release of rubber particles resulting from tire wear.

### 2.2. Cytotoxicity of PM_10_ Organic Extracts

**Figure 2:**
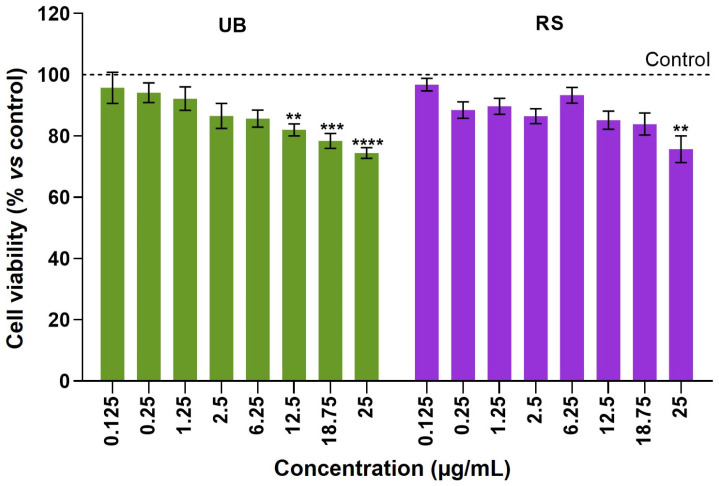
Cell viability of A549 cells after 72-hour incubation with PM_10_ organic extracts from urban-background (UB) and roadside (RS) sites, as assessed using the MTT assay. Bars represent the experimental mean ± standard deviation of three independent experiments with five technical replicates each. Asterisks indicate statistical significance relative to control (Dunnett’s test, ** *p* < 0.01, *** *p* < 0.005, **** *p* < 0.001). The dotted line represents cell viability in untreated controls (100%), to which exposed cells were compared.

The effects of the PM_10_ organic extracts (OC concentration range from 0.125 μg/mL to 25 μg/mL) on the viability of human epithelial cells were investigated with the MTT assay and presented as a percentage of viability relative to unexposed controls (Figure 2).

The A549 lung cells displayed a dose-dependent decrease in viability upon 72 h exposure to UB and RS PM_10_ samples. At the highest OC concentration tested (25 µg/mL), UB and RS extracts significantly decreased cell viability, respectively, to 74.5 ± 6.4% and 75.7 ± 13.8%, relative to control cells. At lower concentrations, the UB PM_10_ extract caused significant differences to control cells at concentrations ≥ 12.5 µg/mL, while the RS PM_10_ samples caused milder, non-significant decreases in cell viability. Based on these results, OC concentrations of 12.5 and 25 µg/mL were selected for subsequent assays. These results are in line with our previous studies that showed concentration-dependent decreases in the viability of A549 cells upon exposure to PM_10_ extracts obtained from emissions related to cooking and ironing activities [46], biomass burning [47], wood-burning appliances [48], and residential pellet combustion [49].

**Figure 3:**
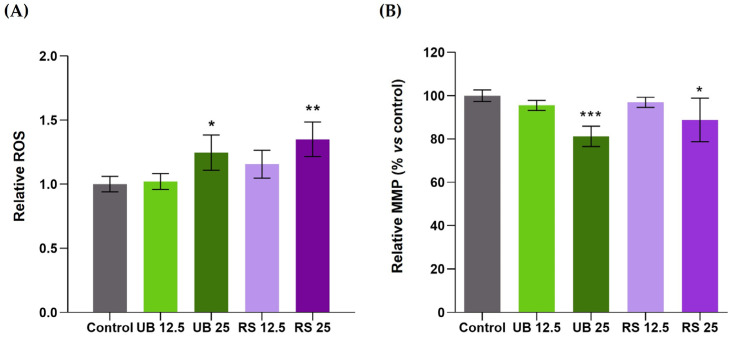
Effects of 72-h exposure of A549 cells to PM_10_ organic extracts from urban background (UB) and roadside (RS) sites, at OC concentrations of 12.5 and 25 µg/mL, on (**A**) intracellular ROS levels and (**B**) mitochondrial membrane potential (MMP). Bars represent the experimental mean ± standard deviation. Asterisks indicate statistical significance relative to control (Dunnett’s test, * *p* < 0.05, ** *p* < 0.01, *** *p* < 0.005).

To assess the involvement of oxidative stress in cellular responses to PM_10_, the variation in ROS levels was examined in exposed cells and compared to unexposed controls (Figure 3A). Exposure to both PM_10_ extracts (UB and RS) at the highest concentration tested (25 µg/mL) induced a moderate but significant increase in intracellular ROS levels (Figure 3A). At the lowest concentration (12.5 µg/mL), a slight increase was also detected in RS-exposed cells, although not reaching statistical significance.

The induction of ROS generation is a well-recognized component of PM toxicity [50] and has been frequently reported to occur in lung cells exposed to PM_2.5_ [51,52,53] and to PM_10_ [54,55,56]. For instance, an organic extract of PM_10_ collected in the Brazilian Amazon caused concentration- and time-dependent increases in A549 intracellular ROS [54]. The maximum increase (over 10-fold relative to unexposed controls) was observed at the highest concentration (400 µg/mL) and the longest exposure time (72 h). For 200 µg/mL and 24 h exposure, ROS levels increased 2- to 4-fold, while cell viability remained unaltered. Such ROS production was much higher than that observed in the present study, which may be related to several factors, such as the concentration and the specific chemical composition of the tested PM_10_ extracts. PAHs are amongst PM-bound organic compounds with known pro-oxidant activity, as their metabolic products may participate in redox reactions and generate ROS like hydrogen peroxide and superoxide anions [57]. The PM_10_ extracts assessed in this study contained PAHs with reportedly high pro-oxidant activity such as benzo[a]pyrene and other HMW PAHs. In particular, the RS extract was richer in these compounds than the UB extract, which may contribute to its slightly higher pro-oxidant activity. As ROS accumulation may induce mitochondrial membrane depolarization and impaired mitochondrial function [58], we have also assessed the impact of PM_10_ extracts on the mitochondrial membrane potential (MMP) of A549 cells. The results showed that both PM_10_ extracts at the highest concentration induced significant decreases in MMP, namely to 81 and 88% of control cells, for UB and RS, respectively (Figure 3B). 

**Figure 4:**
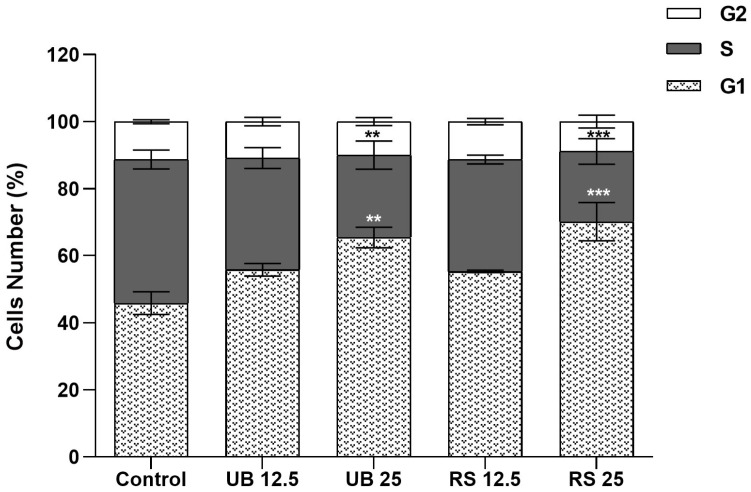
Impact on the cell cycle of 72-hour exposure of A549 cells to PM_10_ organic extracts from urban-background (UB) and roadside (RS) sites, at OC concentrations of 12.5 and 25 µg/mL. Bars represent the experimental mean ± standard deviation. Asterisks indicate statistical significance relative to control (Dunnett’s test, ** *p* < 0.01, *** *p* < 0.005).

Our results further showed that PM_10_ organic extracts interfered with A549 cell cycle dynamics (Figure 4). Exposure to the highest concentrations of UB and RS PM_10_ extracts induced an increase in the % of cells at the G0/G1 phase, together with a decrease in the % of cells at the S phase. These changes are suggestive of cellular stress and DNA damage, as cell cycle arrest at G0/G1 represents a common mechanism to prevent mutagenesis [59]. Reyes-Zarate and co-authors (2016) also demonstrated a G0/G1 cell cycle arrest in A549 cells exposed to a sub-lethal concentration of PM_10_ and related it to activation of signal transducers and activators of transcription-3 (STAT3) and, consequently, apoptosis evasion [60]. Moreover, G0/G1 cycle arrest and formation of DNA strand breaks were reported as early events in the response of A549 cells to 400 µg/mL of a PM_10_ organic extract [54]. 

### 2.3. Impact of PM_10_ Organic Extracts on the Intracellular Metabolic Profile

**Figure 5:**
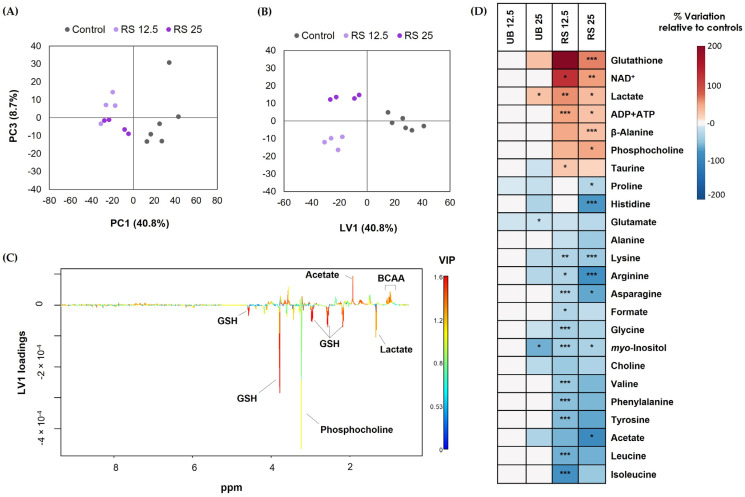
Metabolic effects on A549 cells upon 72-hour exposure to PM_10_ organic extracts from urban-background (UB) and roadside (RS) sites, at OC concentrations of 12.5 and 25 µg/mL. Score scatter plots were obtained using (**A**) PCA and (**B**) PLS-DA of NMR profiles recorded for control and RS-exposed samples, along with (**C**) PLS-DA LV1 loadings; (**D**) Heatmap summarizing the metabolite variations in the aqueous extracts of exposed cells relative to unexposed controls. Data represents the mean of 4 to 6 replicates. The statistical significance is indicated: * *p* < 0.05; ** *p* < 0.01; *** *p* < 0.005.

^1^H NMR analysis of cells’ aqueous extracts enabled the identification of over 30 intracellular metabolites, including carboxylic acids (e.g., lactate and acetate), several amino acids and derivatives, glutathione (GSH), choline-containing compounds, and nucleotides (Supplementary Figure S1 and Table S2). As a first approach to assess the impact of PM_10_ exposure on the cells’ metabolic profile, multivariate analysis was applied to the ^1^H NMR spectra. When cells were exposed to the UB PM_10_ sample, there was no clear separation between control and exposed groups in PCA, indicating a weak impact on cellular metabolism. On the other hand, for RS-exposed samples, the PCA score scatter plot clearly separated control and exposed groups along PC1 (40.8% of variance explained) (Figure 5A). Subsequent PLS-DA confirmed this separation, while additionally discriminating between the two exposure concentrations (Figure 5B). Representation of the LV1 loadings colored by variable importance to the projection (VIP) further revealed the most striking metabolic differences between control and exposed cells (Figure 5C). 

The magnitude and statistical significance of variations in exposed vs. control cells were then assessed using spectral integration of the metabolite signals. The results are summarized in the form of a heatmap (Figure 5D) and presented in detail in Supplementary Table S3. The metabolic profile of cells exposed to the UB PM_10_ organic extract was only mildly affected at the high-exposure concentration, with statistically significant differences being observed for three metabolites. These were lactate (increased in exposed cells), glutamate, and *myo*-inositol (decreased in exposed cells). Moreover, variations not reaching statistical significance but of reasonably large magnitude (|ES| > 0.8) included increases in GSH and decreases in some amino acids. As for the RS PM_10_ extract, it extensively impacted the metabolic composition of lung cells, triggering differences in the levels of 25 intracellular metabolites. These comprised increases in GSH, NAD^+^, lactate, ADP and ATP, beta-alanine, phosphocholine and taurine, along with decreases in choline, acetate, formate, *myo*-inositol, and 13 amino acids. 

The metabolic signature induced by the RS PM_10_ extracts in lung cells suggests interference with central metabolic pathways directly involved in energy generation. Pronounced changes included increases in intracellular lactate, NAD^+^, and ADP and ATP. These changes suggest that cells increased the conversion of glycolysis-derived pyruvate into lactate, a reaction that regenerates NAD^+^ and sustains glycolytic activity and substrate-level energy production. At the same time, decreases in several amino acids suggest their anaplerotic use to fuel the TCA cycle, likely to compensate for the reduced availability of pyruvate-derived acetyl-CoA, and to support other processes, such as GSH synthesis.

The overall increase in ADP and ATP is an indicator of low cytotoxicity, in agreement with the relatively high levels of cell viability measured by MTT (>75%) and could reflect the protective role of metabolism in avoiding severe toxicity at mild exposure concentrations. Indeed, while highly cytotoxic PM concentrations were reported to impair mitochondrial respiration and energy production [39,61,62,63,64,65], there is evidence that PM exposure at sub-toxic doses can upregulate lung cellular metabolism [66,67,68]. For instance, exposure of human bronchial epithelial cells BEAS-2B to low, non-apoptotic concentrations of fine PM led to increased mitochondrial mass and OXPHOS, a response that was associated with the upregulation of the nuclear factor erythroid 2 p45-related factor 2 (NRF2) [66]. This transcription factor regulates the cellular antioxidant defense system and can be activated under low levels of oxidative stress to protect cells from harmful pro-oxidant effects [63]. However, in the present study, the observed decrease in MMP suggests some degree of mitochondrial dysfunction, which could negatively affect ATP synthesis through OXPHOS. Hence, the observed maintenance or even enhancement of ATP levels in PM10-exposed cells is more likely to arise from stimulation of glycolytic energy production, as corroborated by increased intracellular levels of lactate and NAD^+^. 

Our results additionally showed a significant increase in the intracellular levels of GSH, which is a ubiquitous endogenous antioxidant, with a key role in maintaining the oxidative balance in lung epithelial cells and in controlling lung inflammatory processes [69]. Indeed, studies have demonstrated the highly protective effects of intracellular GSH against toxicity induced in lung cells by different agents, such as copper [70] and gold nanoparticles [71]. Furthermore, in another work, ROS levels in PM-exposed A549 lung cells were found to be inversely correlated with the total antioxidant capacity, to which GSH is a major contributor [53]. In the present study, ROS levels moderately increased upon exposure to the higher concentrations of UB and RS PM_10_ extracts. GSH increased in both cases, although the difference to controls was only statistically significant in the case of RS-exposed samples (Figure 5D). In future work, it would be interesting to measure ROS and GSH levels at shorter incubation times, in order to assess the temporal dynamics of their correlation. 

Other PM_10_-induced metabolic effects comprised: (i) variations in choline-containing compounds (possibly related to membrane turnover); (ii) decreases in acetate (precursor of acetyl-CoA) and formate (source of one-carbon units and precursor of purine biosynthesis); (iii) accumulation of β-alanine (precursor of pantothenate, required for CoA biosynthesis) and of taurine (sulfur-containing amino acid with membrane-stabilizing and antioxidant properties [72].

## 3. Materials and Methods

### 3.1. PM_10_ Sampling

Samples of atmospheric PM_10_ were collected at two sites of a mid-size city in the centre of Portugal (Coimbra): a residential area classified as an urban-background (UB) site, and a traffic-impacted-location classified as roadside (RS). The detailed description of these locations and of the sampling campaign was published elsewhere [73]. In brief, at each site, a high-volume air sampler (MCV CAV-A/mb, Barcelona, Spain) equipped with pre-fired 15 cm diameter quartz fibre filters (Pall Corporation, Port Washington, NY, USA)—running at a flow rate of 30 m^3^ h^−1^—was employed to collect PM_10_ samples. Sampling was performed for 24 h, every two days, over six months. For the present study, three filters from the UB site and three filters from the RS location, collected on the same days during the winter season, were used to prepare PM_10_ extracts representative of each site.

### 3.2. Chemical Characterization of PM_10_ Samples

The organic carbon (OC) content in 9 mm punches of the PM_10_-loaded filters was quantified using a thermo-optical transmission technique, following the EUSAAR2 protocol [74]. For assessing the composition in PAHs and plasticizers, organic extracts from 47 mm filter punches were analyzed using gas chromatography coupled to mass spectrometry with a GC-MS spectrometer (model QP5050A, Shimadzu, Kyoto, Japan) equipped with a TRB-5MS 30 m × 0.25 mm × 0.25 μm column. The analysis was accomplished by single ion monitoring (SIM) using a mixture of deuterated internal standards. Further details can be found in Alves and co-authors (2021) [75]. 

### 3.3. PM_10_ Extraction and Sample Preparation for Biological Assays

PM_10_-loaded filter punches (47 mm in diameter) were sequentially extracted by adding dichloromethane (125 mL, 24 h reflux) and methanol (2 additions of 25 mL; 10 min in ultrasonic bath each). The resulting fractions were added together, filtered, and concentrated to a volume lower than 1 mL using a TurboVap concentrator (Biotage Charlotte, NC, USA). Then, the samples were dried under a nitrogen flow, sealed up in glass vials, and preserved at −20 °C. 

For biological assays, dried PM_10_ organic extracts were combined to provide representative samples of the UB and RS sites by reconstitution in dimethyl sulfoxide (DMSO, Sigma Aldrich St. Louis, MO, USA). To obtain sufficiently large volumes for all planned assays, the stock solutions were prepared at a total OC concentration of 2.5 mg/mL. Serial dilutions in complete culture medium were then performed. The highest concentration tested was 25 µg/mL, to restrain the maximum DMSO concentration to 1%, which was confirmed to be non-toxic, in agreement with previous results by Alves and co-authors (2023) [76].

### 3.4. Cell Culture

The human lung adenocarcinoma A549 cell line (American Type Culture Collection, Rockville, MD, USA), which displays characteristics of alveolar epithelial cells, was used in this study. Cells were maintained in Kaighn’s Modification of Ham’s F-12 Medium (F-12K), supplemented with 10% (*v*/*v*) of FBS (Fetal Bovine Serum), 1% of penicillin-streptomycin, and 1% fungizone (all from Gibco, Life Technologies, Grand Island, NY, USA) at 37 °C in a humidified incubator with 5% CO_2_. Cell confluence and morphology were routinely observed under an inverted microscope (Nikon^®^Eclipse TS100, Nikon, Tokyo, Japan) and subculture was performed when cells reached 80–90% confluence. 

### 3.5. Cell Viability Assay

The cytotoxicity of PM_10_ organic extracts towards A549 cells was assessed by the MTT ([3-(4, 5-dimethylthiazol-2-yl)-2,5-diphenyltetrazolium bromide]) assay. Cells were seeded at a density of 1.5 × 10^4^ cells/mL in 96-well plates and incubated for 24 h to promote cell adhesion. Then, cells were treated with eight different concentrations of PM_10_ organic extracts in the range of 0.125–25 µg/mL for 72 h. Control cells were treated with culture medium containing 1% DMSO (the maximum concentration present in PM_10_ extracts). After 72 h, 50 μL of MTT (1.0 mg/mL in PBS) was added to each well and incubated for 4 h at 37 °C. Then, the MTT solution was removed and replaced by 150 μL of DMSO, and the plate was shaken in the dark for 2 h at room temperature to dissolve the formazan crystals. The absorbance of the samples was measured using a microplate reader (Synergy HT^®^ Multi-Mode; BioTek^®^, Vinooski, VT, USA) at 570 nm with blank corrections. Three independent experiments were carried out, each with five technical replicates.

### 3.6. Quantification of Intracellular ROS

The intracellular ROS levels were assessed using flow cytometry with the fluorogenic dye 2′,7′-dichlorodihydrofluorescein diacetate (DCFH-DA, Merck KGaA, Darmstadt, Germany), a cell-permeable compound that is cleaved intracellularly and oxidized by ROS to fluorescent 2′,7′-dichlorofluorescein (DCF). Cells were seeded at a density of 1.5 × 10^4^ cells/mL in 12-well plates and incubated for 24 h. Then, cells were exposed to UB and RS PM_10_ extracts for 72 h at OC concentrations of 12.5 and 25 µg/mL. Control cells were incubated with 1% DMSO in culture medium. After this period, the medium was removed and the cells were washed with 500 μL of PBS (pH 7.2), followed by a 30 min incubation with 500 μL of DCFH-DA 10 μM in culture medium supplemented with 2% FBS. Finally, cells were washed, trypsinized, and resuspended in cold medium containing 2% FBS for analysis on an Attune^®^ Acoustic Focusing Cytometer (Applied Biosystems, Thermo Fischer Scientific, Waltham, MA, USA), within 45 min after cell collection. Data analysis was performed using the FlowJo software version 10.7.1 (Tree Star Inc., Ashland, OR, USA).

### 3.7. Mitochondrial Membrane Potential (MMP)

Changes in MMP were assessed using flow cytometry with the cationic fluorescent dye Rhodamine 123 (Rho123). This dye selectively accumulates in healthy mitochondria with high negative membrane potential, while a decrease in its uptake—and hence fluorescence intensity—can be associated with a decrease in the negative charge across the inner mitochondrial membrane. For this assay, 1.5 × 10^4^ cells/mL were seeded in 6-well plates and, after cell adhesion, they were exposed for 72 h to the UB and RS PM_10_ extracts, as described above. At the end of the exposure time, cells were incubated with 5 µg/mL of Rho123 (Sigma-Aldrich, St. Louis, MO, USA) for 30 min. Then, cells were washed with PBS, trypsinized, and centrifuged at 700× *g* for 5 min at 4 °C. After that, the cell pellet was washed with 1 mL of PBS containing 1% BSA and resuspended in 500 µL of PBS containing 1% BSA and 5 µg/mL PI (Sigma-Aldrich, St. Louis, MO, USA). Samples were then analyzed using flow cytometry with an Attune^®^ Acoustic Focusing Cytometer (Applied Biosystems, Thermo Fischer Scientific, Waltham, MA, USA) and at least 10,000 events were analyzed. The loss of MMP was visualized as indicated by the reduction in Rho123 FL.

### 3.8. Cell Cycle Analysis

Cells were seeded at a density of 1.5 × 10^4^ cells/mL in 12-well plates and incubated for 24 h, after which they were exposed for 72 h to the UB and RS PM_10_ extracts, as described above for the ROS assay. Cells were then washed with 500 μL of PBS, trypsinized, resuspended in 300 μL of culture medium, and centrifuged at 700× *g* for 5 min. The resulting cell pellet was then fixed with 1 mL of cold ethanol 85% and stored at −20 °C until analysis. At the time of analysis, cells were centrifuged at 112× *g* for 6 min at 4 °C and resuspended in 800 μL of PBS, followed by filtration through a nylon membrane (41 µm). Each sample was then incubated for 10 min with 50 μg/mL of ribonuclease A (RNAse, Sigma-Aldrich, St. Louis, MO, USA), and 50 μg/mL of propidium iodide (PI, ≥94%; Sigma-Aldrich, St. Louis, MO, USA), for additional 30 min, at room temperature in the dark. Analysis of cell cycle was performed on an Attune^®^ Acoustic Focusing Cytometer flow cytometer (Applied Biosystems, Thermo Fischer Scientific, Waltham, MA, USA). For each sample, at least 5000 events were acquired. The FlowJo software (FlowJo LLC, Ashland, OR, USA) was employed to determine the percentage of cells at Gap 0 (G0)/Gap 1 (G1), Synthesis (S), and Gap 2 (G2)/Mitosis (M) phases of the cell cycle. Two independent assays, each with two replicates, were performed for each treatment.

### 3.9. NMR Metabolomics

Cells were seeded in 100 mm diameter dishes at a density of 4 × 10^4^ cells/mL and incubated for 24 h to allow for cell attachment. Then, cells were exposed to UB and RS PM_10_ at OC concentrations of 12.5 and 25 µg/mL for 72 h. Control cells were incubated with 1% DMSO in culture medium. Four to six replicates per condition were collected within three independent assays.

At the end of the 72-hour exposure period, the culture medium was removed, cells were washed with cold PBS and were extracted using a dual-phase extraction, with methanol/chloroform/water (1:1:0.7) as previously described in detail [77]. Aqueous extracts were then dried in a vacuum concentrator (CentriVap, model 73100, Labconco, Kansas City, MO, USA) and stored at −80 °C. For NMR analysis, cell extracts were reconstituted in 600 µL of deuterated PBS (100 mM, pH 7.4) containing 0.1 mM trimethylsilylpropanoic acid (TSP-d_4_) and transferred into 5 mm NMR tubes. 

Spectra acquisition was carried out in a Bruker Avance III HD 500 NMR spectrometer (University of Aveiro, PTNMR Network), operating at 500.13 MHz for ^1^H observation. Standard ^1^H 1D spectra with water suppression (pulse program “noesypr1d”) were acquired with a 7002.8 Hz spectral width, 32 k data points, 2 s relaxation delay, and 512 scans. Spectral processing was performed in TopSpin 4.0.3 (Bruker BioSpin, Rheinstetten, Germany) and comprised cosine multiplication (ssb 2), zero-filling to 64 k data points, manual phasing, baseline correction, and chemical shift calibration to the TSP signal at 0 ppm. The 2D ^1^H-^1^H total correlation (TOCSY) spectra and J-resolved spectra were also acquired for selected samples to aid metabolite assignment, based on matching spectral information to reference spectra available in Chenomx (Edmonton, AB, Canada) and Bruker BBIOREFCODE (Amix-Viewer version 3.9.15, Bruker BioSpin), Rheinstetten, Germany. 

For multivariate analysis, carried out in SIMCA-P 11.5 (Umetrics, Umeå, Sweden), spectral matrices were normalized to total area and scaled to unit variance. The results of Principal Component Analysis (PCA) and Partial Least Squares Discriminant Analysis (PLS-DA) were represented as score scatter plots and corresponding loadings plots colored by variable importance to the projection (VIP). Signal integration was performed in Amix-Viewer and the variation in selected metabolites in treated vs. control samples was assessed, along with the effect size (ES) and statistical significance (*p*-value). Metabolite variations of larger magnitude (|ES| > 0.8) were depicted in a heatmap, generated using the R software version 4.1.3 (R Core Team (2020). R: a language and environment for statistical computing. R Foundation for Statistical Computing, Vienna, Austria. URL http://www.R-project.org/ (accessed on 30 September 2023)).

### 3.10. Statistical Analysis

Statistical analysis was performed in GraphPad Prism (GraphPad Software version 9, Inc., La Jolla, CA, USA). One-way analysis of variance (ANOVA) with Dunnett’s test (*p* ≤ 0.05) was used for multiple comparisons. Statistical differences are indicated as * *p* < 0.05, ** *p* < 0.01, *** *p* < 0.005, **** *p* < 0.001.

## 4. Conclusions

Following a 72-hour exposure to PM_10_ extracts at concentrations that exhibited low cytotoxicity (cell viability exceeding 75%), lung A549 cells displayed increased ROS generation, decreased MMP, arrest of the cell cycle at the G0/G1 phase, and modifications in the intracellular metabolic profile. Notably, the PM_10_ extract derived from the RS location—which contained a higher concentration of particularly hazardous PAHs and plasticizers—induced more pronounced metabolic alterations compared to the UB extract. The observed metabolic shifts suggest that PM_10_ exposure triggers a strengthening of antioxidant defenses and an upregulation of energy production pathways, a response that likely reflects the protective role of metabolic adaptations in mitigating the onset of severe cytotoxic effects.

While informative, this study has some noteworthy limitations. Firstly, the chemical characterization of the PM_10_ extracts was not exhaustive. Although PAHs and plasticizers are well-recognized as highly toxic organic constituents of PM, the influence of other unanalyzed chemicals cannot be excluded. Secondly, while A549 cells represent a widely accepted in vitro model for human pulmonary epithelial cells, it is essential to acknowledge that the effects observed in vitro may not fully mirror the complexities of in vivo scenarios. Moreover, it is important to recognize that metabolomics offers a static snapshot of the metabolites present, which might not accurately reflect the dynamic changes that occur in vivo, and can only partly reveal the modulation of metabolic pathways. Hence, additional strategies like integrating metabolomics data with proteomics and/or transcriptomics, as well as assessing the expression of selected metabolic genes and proteins, should be explored in future studies to provide more comprehensive mechanistic insights. 

## Data Availability

The data supporting reported results are available from the corresponding author upon reasonable request.

## Supplementary Materials

The supporting information can be downloaded at: https://www.mdpi.com/article/10.3390/ijms242316896/s1.
