# Supplementary material for: Biological Impact of Organic Extracts from Urban-Air Particulate Matter: An In Vitro Study of Cytotoxic and Metabolic Effects in Lung Cells
Source: Int J Mol Sci. 2023 Nov 29;24(23):16896. doi: 10.3390/ijms242316896 (PMC10706705; doi:10.3390/ijms242316896)
Supplement: Supplementary file 1 [file ijms-24-16896-s001.zip › ijms-2667815-supplementary.pdf]

Supplementary Material

Biological impact of organic extracts from urban-air particulate matter: an *in vitro* study of cytotoxic and metabolic effects in lung cells

Tatiana D. Silva[1,2], Célia Alves[3], Helena Oliveira[2] and Iola F. Duarte[1,*]

[1] Department of Chemistry. CICECO – Aveiro Institute of Materials. University of Aveiro. 3810-193 Aveiro. Portugal; ioladuarte@ua.pt (I.F.D.); tatiana.silva@ua.pt (T.D.S.)

[2] Department of Biology. CESAM - Centre for Environmental and Marine Studies. University of Aveiro. 3810-193 Aveiro. Portugal; holiveira@ua.pt (H.O.)

[3] Department of Environment and Planning. CESAM - Centre for Environmental and Marine Studies. University of Aveiro. 3810-193 Aveiro. Portugal; celia.alves@ua.pt (C.A.)

* Correspondence: ioladuarte@ua.pt

Figures

Figure S1. Representative ^{1}H NMR spectrum of A549 lung cells.

Tables

Table S1. Chemical characterization of the PM_{10}-loaded filters collected at two locations in Coimbra - urban background (UB) and roadside (RS) – and used in this work to assess the biological effects on lung cells.

Table S2. Metabolites identified in the A549 lung cells based on 1D and 2D spectral data.

Table S3. Metabolite variations in the aqueous extracts of A549 lung cells exposed for 72h to UB and RS PM_{10} organic extracts in relation to unexposed controls.

Figure S1. Representative ^1H NMR spectrum of A549 lung cells with some assignments indicated (numbers identified in Table S2).

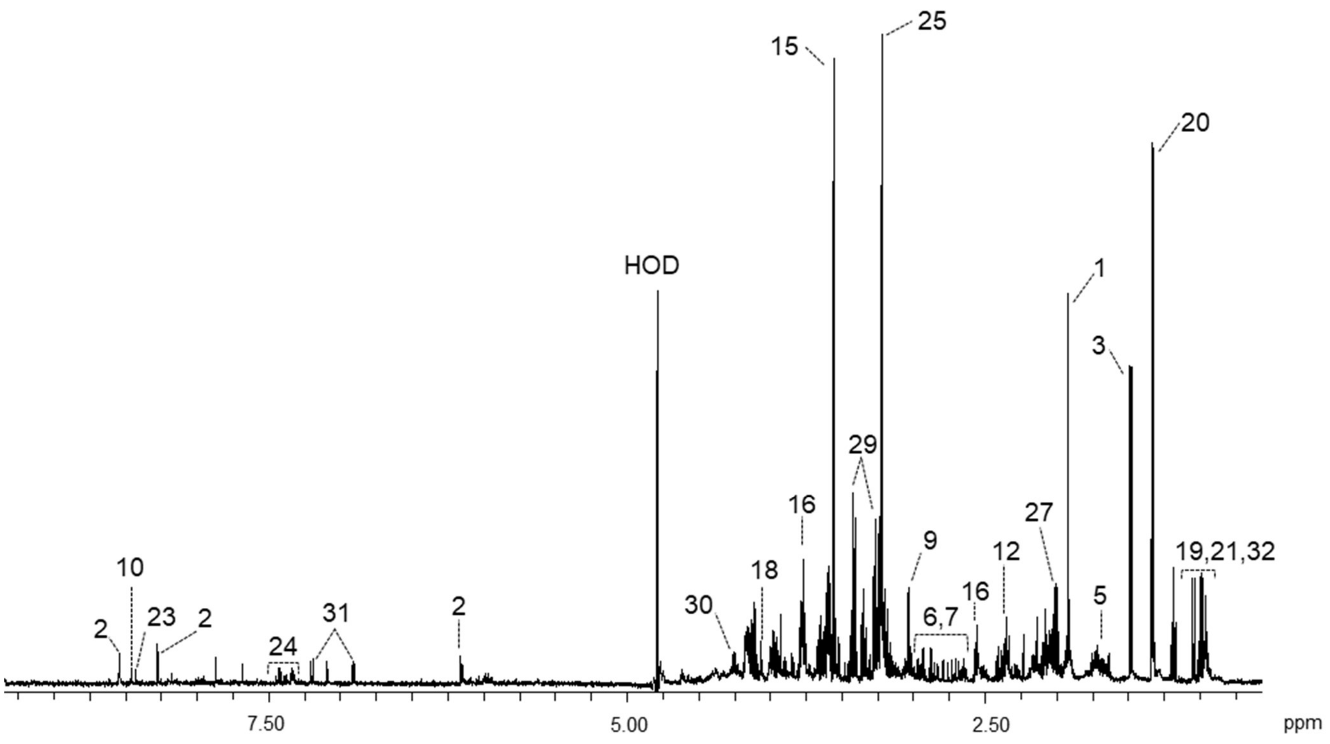

Table S1. Chemical characterization of the PM_{10}-loaded filters collected at two locations in Coimbra - urban background (UB) and roadside (RS) – and used in this work to assess the biological effects on lung cells.

	**PM_{10} – Urban Background (UB)**			**PM_{10} – Roadside (RS)**		
**Filter**	**UB1**	**UB2**	**UB3**	**RS1**	**RS2**	**RS3**
PM_{10} concentration (μg/cm^2)						
	168.76	267.40	137.16	214.70	370.62	170.19
**Organic Carbon** (OC) *(μg/cm^2)*						
	42.88	72.05	34.02	47.68	86.71	52.84
PAHs (ng/cm^2)						
Benzothiazole	0.98	5.27	5.62	0.75	0.90	0.90
Carbazole	0.13	0.45	0.15	0.16	0.39	0.11
p-Terphenyl	0.09	0.47	0.15	0.19	0.47	0.14
Retene	4.08	19.74	5.16	4.56	29.52	7.83
Naphthalene	7.24	15.82	17.02	7.38	8.53	10.96
C1-naphthalenes	5.95	3.64	3.92	5.22	6.20	6.53
C2-naphthalenes	4.09	4.75	3.12	3.93	6.41	4.09
C3-naphthalenes	2.54	2.21	1.10	2.29	3.81	2.60
C4-naphthalenes	0.67	4.57	0.97	1.84	6.85	1.57
Acenaphthylene	0.17	1.15	0.97	0.33	0.37	0.47
Acenaphthene	0.99	1.24	0.86	1.08	1.19	0.98
Fluorene	6.79	3.27	3.85	5.62	6.55	6.48
C1-fluorenes	5.23	45.31	10.44	7.92	30.86	7.20
C2-fluorenes	13.53	29.70	3.81	21.97	35.88	4.82
Phenanthrene	0.77	3.08	2.08	1.49	4.84	2.49
C1-phenanthrenes	1.17	7.60	1.80	2.48	10.79	2.15
Anthracene	0.10	0.75	0.35	0.33	0.99	0.48
Fluoranthene	1.31	7.97	3.28	2.40	9.83	3.69
C1-fluoranthenes/pyrenes	1.98	12.38	3.45	3.45	10.48	4.11
C2-fluoranthenes/pyrenes	0.30	2.23	0.55	0.74	2.40	0.37
Pyrene	1.88	9.90	4.55	3.18	11.77	4.97
Chrysene [a]	1.91	11.43	5.31	5.44	18.47	6.33
C1-chrysenes	1.73	8.48	3.45	4.40	13.62	4.19
C2-chrysenes	0.20	1.84	0.73	0.96	3.46	0.78
C3-dibenzothiophenes	3.08	17.55	5.48	7.99	21.28	9.71
C4-dibenzothiophenes	5.59	16.54	5.81	5.42	22.75	4.91
Benzo[a]anthracene[a]	1.05	7.69	3.77	3.88	12.30	4.03
7,12-Dimethylbenz[a]anthracene	1.38	4.86	1.96	2.23	9.56	3.43
Benzo[b]fluoranthene[a]	2.46	10.52	5.08	4.74	16.59	7.74
Benzo[k]fluoranthene[a]	2.43	9.19	5.68	4.98	15.35	7.33
Benzo[e]pyrene	1.96	7.8	4.18	3.85	11.70	6.08
Benzo[a]pyrene[a]	1.80	8.32	4.34	4.18	14.42	6.66
Perylene	0.81	3.64	1.88	2.07	6.12	2.92

Table S1. (continued)

	PM_{10} – Urban Background (UB)			PM_{10} – Roadside (RS)		
**Filter**	**UB1**	**UB2**	**UB3**	**RS1**	**RS2**	**RS3**
PAHs (ng/cm²)						
Indeno[1,2,3-cd]pyrene [a]	2.31	7.47	4.11	4.36	11.78	5.22
Dibenzo[a,h]anthracene [a]	0.49	1.25	0.61	0.59	1.78	0.79
Benzo[ghi]perylene	2.28	6.71	3.90	4.15	10.16	5.71
Total PAHs	*89.47*	*304.79*	*129.49*	*136.55*	*378.37*	*148.77*
Plasticizers (ng/m³)						
Dimethyl phthalate	0.13	0.00	0.00	0.28	0.88	0.80
Diethyl phthalate	1.81	0.65	0.63	0.88	1.80	1.05
Diisobutyl phthalate	1.75	5.93	6.14	3.48	3.90	4.10
Di-n-butyl phthalate	5.82	8.44	6.07	10.62	15.45	9.61
Benzyl butyl phthalate	0.00	0.00	0.00	2.04	0.00	0.00
Bis(2-ethylhexyl)adipate	1.06	2.64	1.79	2.74	3.37	2.21
Bis(2-ethylhexyl)phthalate	23.34	40.03	19.17	53.39	64.26	24.98
Di-n-octyl phthalate	10.54	0.27	0.83	22.29	15.91	1.02
Total Plasticizers	*44.45*	*57.96*	*34.63*	*95.72*	*105.57*	*43.77*

Legend: [a] Carcinogenic PAHs (IARC)

Table S2. Metabolites identified in the A549 lung cells based on 1D and 2D spectral data.

No.	Compound	δ ^{1}H in ppm (multiplicity)
1	Acetate	1.92 (s)
2	ADP + ATP	4.23 (m); 4.39 (m); 4.59 (m); 4.85 (m); 6.16 (d); 8.28 (s); 8.53 (s)
3	Alanine	1.49 (d); 3.78 (q)
4	β-Alanine	2.57 (t); 3.19 (t)
5	Arginine	1.70 (m); 1.92 (m); 3.26 (t); 3.78 (t)
6	Asparagine	2.88 (dd); 2.94 (dd), 3.99 (dd)
7	Aspartate	2.68 (dd); 2.81 (dd); 3.89 (dd)
8	Choline	3.21 (s); 3.53 (m); 4.07 (m)
9	Creatine	3.04 (s); 3.94 (s)
10	Formate	8.46 (s)
11	Fumarate	6.52 (s)
12	Glutamate	2.08 (m); 2.13 (m); 2.35 (m); 3.77 (dd)
13	Glutamine	2.15 (m); 2.46 (m); 3.77 (t)
14	Glycerophosphocholine	3.24 (s); 3.68 (m); 3.91 (m); 4.32 (m)
15	Glycine	3.57 (s)
16	Glutathione (GSH)	2.17 (m); 2.55 (m); 2.95 (m); 3.80 (m); 4.57 (m)
17	Histidine	3.23 (dd); 3.31 (dd); 4.01 (dd); 7.15 (s); 8.02 (s)
18	*myo*-Inositol	3.30 (t); 3.55 (dd); 3.63 (t); 4.08 (t)
19	Isoleucine	0.94 (t); 1.01 (d); 1.26 (m); 1.48 (m); 1.98 (m); 3.67 (d)
20	Lactate	1.33 (d); 4.12 (q)
21	Leucine	0.96 (t); 1.771 (m); 3.78 (m)
22	Lysine	1.48 (m); 1.73 (m); 1.91 (m); 3.03 (t); 3.79 (t)
23	NAD^{+}	6.04 (d); 6.10 (d); 8.18 (s); 8.20 (m); 8.42 (s); 8.84 (d); 9.15 (d); 9.35 (s)
24	Phenylalanine	3.13 (m); 3.28 (dd); 4.01 (m); 7.33 (d); 7.38 (d); 7.43 (t)
25	Phosphocholine	3.23 (s) ; 3.60 (m) ; 4.16 (m)
26	Phosphocreatine	3.05 (s); 3.96 (s)
27	Proline	2.00 (m); 2.08 (m); 2.35 (m); 3.36 (t); 3.41 (t); 4.14 (t)
28	Pyroglutamate	2.04 (m); 2.41 (m); 2.50 (m); 4.18 (dd)
29	Taurine	3.28 (t); 3.43 (t)
30	Threonine	1.33 (d); 3.61 (d); 4.25 (m)
31	Tyrosine	3.07 (m); 3.20 (m); 3.94 (m); 6.91 (d); 7.20 (d)
32	Valine	0.99 (d); 1.03 (d); 2.28 (m); 3.62 (d)
33	Methionine	2.14 (m); 2.65 (t); 3.87 (dd)

Multiplicity: s, singlet; d, doublet; dd, doublet of doublets; m, multiplet; q, quartet; t, triplet. ADP/ATP, adenosine di/triphosphate; GSH, reduced glutathione; NAD+, nicotinamide adenine dinucleotide.

Table S3. Metabolite variations in the aqueous extracts of A549 lung cells exposed for 72h to UB and RS PM_{10} organic extracts in relation to unexposed controls.

		UB 12.5	UB 25	RS 12.5	RS 25
**Glutathione**	% Var	0	29.05	208.61	62.76
	±		16.05	6.87	19.32
	ES		1.36	8.73	4.24
	p-value		$7.15x10^{-2}$	$1.80x10^{-7}$	$3.13\ x10^{-3}$
**Uridine nucleotides**	% Var	0	27.01	147.38	56.88
	±		15.43	31.07	37.79
	ES		1.17	2.19	2.05
	p-value		$1.45x10^{-1}$	$5.74x10^{-2}$	$6.68x10^{-2}$
**NAD+**	% Var	0	0	123.74	52.27
	±			14.23	23.27
	ES			3.78	2.68
	p-value			$3.13x10^{-3}$	$1.93x10^{-2}$
**Lactate**	% Var	0	27.89	57.00	31.87
	±		12.13	16.23	16.23
	ES		1.92	2.04	2.01
	p-value		$3.51x10^{-2}$	$5.79x10^{-2}$	$5.51\ x10^{-2}$
**ADP+ATP**	% Var	0	0	48.82	27.65
	±			8.74	11.30
	ES			3.31	2.45
	p-value			$1.43x10^{-2}$	$3.52x10^{-2}$
**Beta-alanine**	% Var	0	0	45.95	28.99
	±			7.47	7.19
	ES			2.73	2.56
	p-value			$8.32x10^{-4}$	$1.31\ x10^{-3}$
**Phosphocholine**	% Var	0	0	38.89	47.86
	±			21.35	20.61
	ES			0.75	1.71
	p-value			$1.71\ x10^{-1}$	$1.09\ x10^{-2}$
**Taurine**	% Var	-8.81	-12.60	24.23	13.57
	±	6.52	4.81	5.69	12.66
	ES	-0.87	-1.37	1.95	0.85
	p-value	$2.09x10^{-1}$	$9.85x10^{-2}$	$5.57x10^{-3}$	$2.47\ x10^{-1}$
**Proline**	% Var	0	-16.72	0	-27.49
	±		6.21		8.68
	ES		-1.38		-1.82
	p-value		$9.69x10^{-2}$		$2.75x10^{-2}$
**Histidine**	% Var	0	-32.05	0	-72.60
	±		11.02		10.67
	ES		-1.42		-2.89
	p-value		$7.45x10^{-2}$		$1.71x10^{-3}$

Table S3. (continued)

		UB 12.5	UB 25	RS 12.5	RS 25
**Glutamate**	% Var	-10.29	-15.51	-12.87	-24.95
	±	6.46	3.43	9.83	12.69
	ES	-1.03	-2.58	-0.85	-1.20
	p-value	1.68×10^{-1}	5.85×10^{-3}	2.21×10^{-1}	1.20×10^{-1}
**Alanine**	% Var	0	0	-15.83	-41.95
	±			12.34	13.95
	ES			-0.95	-1.84
	p-value			2.50×10^{-1}	6.03×10^{-2}
**Lysine**	% Var	0	-23.66	-29.46	-41.77
	±		8.31	7.36	8.29
	ES		-1.43	-2.73	-2.66
	p-value		7.94×10^{-2}	4.15×10^{-3}	5.49×10^{-3}
**Arginine**	% Var	0	-27.62	-30.70	-76.64
	±		9.37	10.28	8.71
	ES		-1.43	-2.23	-3.92
	p-value		8.81×10^{-2}	2.43×10^{-2}	8.11×10^{-4}
**Asparagine**	% Var	0	0	-31.54	-64.87
	±			6.48	14.57
	ES			-2.90	-2.31
	p-value			5.41×10^{-4}	2.23×10^{-2}
**Formate**	% Var	0	0	-32.16	-19.52
	±			13.15	7.81
	ES			-1.68	-1.10
	p-value			3.07×10^{-2}	6.25×10^{-2}
**Glycine**	% Var	0	-14.73	-36.38	-30.89
	±		7.61	7.45	19.14
	ES		-1.15	-3.04	-1.05
	p-value		1.13×10^{-1}	4.47×10^{-4}	2.07×10^{-1}
***myo*-Inositol**	% Var	0	-59.92	-38.06	-34.59
	±		13.10	7.38	9.04
	ES		-1.85	-2.87	-1.75
	p-value		5.27×10^{-2}	1.34×10^{-3}	1.08×10^{-2}
**Choline**	% Var	0	-23.13	-42.90	-31.71
	±		11.07	4.69	13.80
	ES		-1.10	-6.32	-1.54
	p-value		1.34×10^{-1}	1.12×10^{-5}	1.08×10^{-1}
**Valine**	% Var	0	0	-43.03	-55.98
	±			7.77	17.30
	ES			-4.03	-1.88
	p-value			5.98×10^{-4}	6.26×10^{-2}
**Phenylalaline**	% Var	0	0	-51.52	-56.64
	±			10.26	22.29
	ES			-3.22	-1.38
	p-value			2.73×10^{-4}	1.02×10^{-1}

Table S3. (continued)

		UB 12.5	UB 25	RS 12.5	RS 25
**Tyrosine**	% Var	0	0	-54.36	-65.05
	±			9.27	21.85
	ES			-4.44	-1.67
	p-value			$2.44x10^{-4}$	$8.64x10^{-2}$
**Acetate**	% Var	0	-31.67	-58.50	-88.65
	±		8.74	6.96	16.85
	ES		-1.93	-6.56	-2.67
	p-value		$2.01x10^{-2}$	$3.24x10^{-5}$	$2.56x10^{-2}$
**Leucine**	% Var	0	0	-60.14	-59.75
	±			10.98	24.17
	ES			-3.53	-1.32
	p-value			$2.32x10^{-4}$	$1.12x10^{-1}$
**Isoleucine**	% Var	0	0	-76.38	-43.78
	±			18.94	30.02
	ES			-2.79	-0.78
	p-value			$1.18x10^{-3}$	$2.62x10^{-1}$
